# Towards precision medicine in bariatric surgery prescription

**DOI:** 10.1007/s11154-023-09801-9

**Published:** 2023-05-02

**Authors:** Sofia S. Pereira, Marta Guimarães, Mariana P. Monteiro

**Affiliations:** 1 UMIB – Unit for Multidisciplinary Research in Biomedicine, ICBAS – School of Medicine and Biomedical Sciences, University of Porto, Rua Jorge Viterbo Ferreira 228, 4050-313 Porto, Portugal; 2 ITR – Laboratory of Integrative and Translocation Research in Population Health, Rua das Taipas 135, 4050-600 Porto, Portugal; 3 Department of General Surgery, Hospital São Sebastião, Centro Hospitalar de Entre o Douro e Vouga, Rua Dr. Cândido Pinho, 4050-220 Santa Maia da Feira, Portugal

**Keywords:** Bariatric surgery, Weight loss, Outcome predictors, Precision medicine

## Abstract

Obesity is a complex, multifactorial and chronic disease. Bariatric surgery is a safe and effective treatment intervention for obesity and obesity-related diseases. However, weight loss after surgery can be highly heterogeneous and is not entirely predictable, particularly in the long-term after intervention. In this review, we present and discuss the available data on patient-related and procedure-related factors that were previously appointed as putative predictors of bariatric surgery outcomes. In addition, we present a critical appraisal of the available evidence on which factors could be taken into account when recommending and deciding which bariatric procedure to perform. Several patient-related features were identified as having a potential impact on weight loss after bariatric surgery, including age, gender, anthropometrics, obesity co-morbidities, eating behavior, genetic background, circulating biomarkers (microRNAs, metabolites and hormones), psychological and socioeconomic factors. However, none of these factors are sufficiently robust to be used as predictive factors. Overall, there is no doubt that before we long for precision medicine, there is the unmet need for a better understanding of the socio-biological drivers of weight gain, weight loss failure and weight-regain after bariatric interventions. Machine learning models targeting preoperative factors and effectiveness measurements of specific bariatric surgery interventions, would enable a more precise identification of the causal links between determinants of weight gain and weight loss. Artificial intelligence algorithms to be used in clinical practice to predict the response to bariatric surgery interventions could then be created, which would ultimately allow to move forward into precision medicine in bariatric surgery prescription.

## Obesity

Obesity is a complex, chronic and multifactorial disease [1]. Weight loss response to obesity treatment interventions can be highly variable, suggesting that obesity is a heterogeneous disease in which patient specific characteristics are important determinants for treatment effectiveness [2, 3].

Bariatric surgery was proven to be a safe, effective and cost-effective intervention for obesity treatment [4]. Moreover, bariatric surgery remains the most effective for the treatment of obesity and obesity related disorders, by achieving significant and sustained body weight loss along with long-term remission of several comorbidities including type 2 diabetes (T2D), hypertension, sleep apnea and metabolic syndrome [5]. Bariatric surgery is currently recommended for patients with a body mass index (BMI) ≥ 35 or ≥ 30 kg/m^2^ with co-morbidity [6].

Since the 1950s, several different bariatric surgery procedures have been introduced into clinical practice as well as discontinued; nowadays Roux-en-Y Gastric Bypass (RYGB), Mini gastric-bypass, Sleeve gastrectomy (SG) and Biliopancreatic diversion (BPD) with or without Duodenal Switch (DS) are the most frequently performed bariatric surgical interventions, globally [7]. Among these, SG and RYGB represent the largest proportion of bariatric procedures conducted in present-day surgical practice, with a trend for a rising popularity of SG at each passing year [8].

## Weight loss after bariatric surgery

In patients with severe obesity, bariatric surgery is the most effective treatment at achieving sustained weight loss over the long-term [9, 10]. Nevertheless, the weight loss achieved is variable depending on the bariatric surgical technique performed. In the past few years, several meta-analyses have compared the weight loss efficacy of restrictive [SG and adjustable gastric band (AGB)], dysabsortive [BPD with or without DS and single anastomosis duodenal-ileal bypass with sleeve gastrectomy (SADI-S)] and mixed procedures (RYGB and one-anastomosis gastric bypass -OAGB) [11–13]. Based on twenty randomized clinical trials (RCTs), Currie A *et al* found that the percentage of excess weight loss (%EWL) at 1 year after surgery, was not significantly different when patients submitted to RYGB or SG were compared, but patients submitted to OAGB had greater %EWL than those patients that underwent either RYGB or SG. In contrast, the weight loss efficacy 3 and 5 years after surgery was greater after RYGB when compared to SG, while no significant differences in %EWL were observed between OAGB and SG or RYGB [11]. In a meta-analysis with a longer follow-up (> 5 years) including eighty prospective, retrospective or randomized clinical trials (RCTs), Golzarand M *et al* reported that the %EWL was greater in patients submitted to RYGB (62.6%) when compared to SG (53.3%) or adjustable gastric banding (AGB, 47.9%) [12]. Moreover, a meta-analysis comprehending only those studies with data on weight loss over 10 or more years of follow-up, showed a %EWL weighted mean of 45.9% for AGB (17 studies, 8485 patients), 58.3% for SG (2 studies, 163 patients), 56.7% for RYGB (18 studies, 9386 patients) and 74.1% (11 studies, 5074 patients) BPD with or without DS [13]. Even though these results should be analyzed with due caution given the unequal number of studies and patients included for each bariatric procedure. It should be highlighted that although the majority of studies described a greater %EWL after RYGB or OAGB than SG, this procedure continues to be the most frequently performed bariatric surgical procedure worldwide [8].

A recent meta-analysis has also compared the weight loss effectiveness between the two less absorptive surgeries most frequently performed, namely BPD-DS and SADI-S, which found that the %EWL at 2 years after surgery, was greater after BPD-DS when compared to SADI-S. Although, the latter group was associated with fewer long-term (> 30 d) surgical complications [14].

The widespread use of surgeries conducted worldwide over the past decade enabled the availability of a large number of long-term follow-up datasets, and it became clear that there is a considerable proportion of patients with inadequate weight loss—primary non-responders—or experiencing weight regain—secondary non-responders—after surgery. Although, there is a lack of universal definitions for primary and secondary non-responders that impairs an adequate patient classification according to weight loss results, the number of patients that underwent bariatric surgery and seek medical attention for insufficient weight loss continues to increase [15].

Patients generally achieve a maximum weight loss 1 or 2 years after bariatric surgical procedures, but there is a wide interindividual response to the various types of bariatric surgery, particularly during longer-term follow-up [16, 17].

A large prospective cohort of nearly 6000 patients showed that 17.1% of the patients presented inadequate weight loss (< 25% total weight loss) 1 year after RYGB. The same study classified 23.1% of the patients as primary non-responders, 5 years after RYGB, based on the following criteria: % of excess BMI loss < 50%, total weight loss < 20% or BMI > 35 kg/m^2^ where initial BMI was < 50 kg/m^2^, or > 40 kg/m^2^ where initial BMI was > 50 kg/m^2^ [18]. Similarly, a meta-analysis study reported that 27.8% of the 652 patients included in the study that were submitted to SG ≥ 7 years, presented weight recidivism (< 50% EWL) [19].

Ensuring appropriate patient follow-up after bariatric surgery is of utmost importance not only for a timely identification and targeted intervention of any potential postoperative surgical or medical complications, but also to enable body weight monitoring and early intervention whenever there is weight regain [20, 21]. Notwithstanding, the influence of patients’ adherence to follow-up after bariatric surgery on weight loss success is highly debatable. Previous studies found a significant association between patient adherence to a follow-up 0.5 to 3 years after surgery and %EWL [22, 23]. However, no association was found between patient compliance to follow up over 3 years and weight loss after RYGB and SG [23].

## Predictors of success in bariatric surgery

**Fig. 1:**
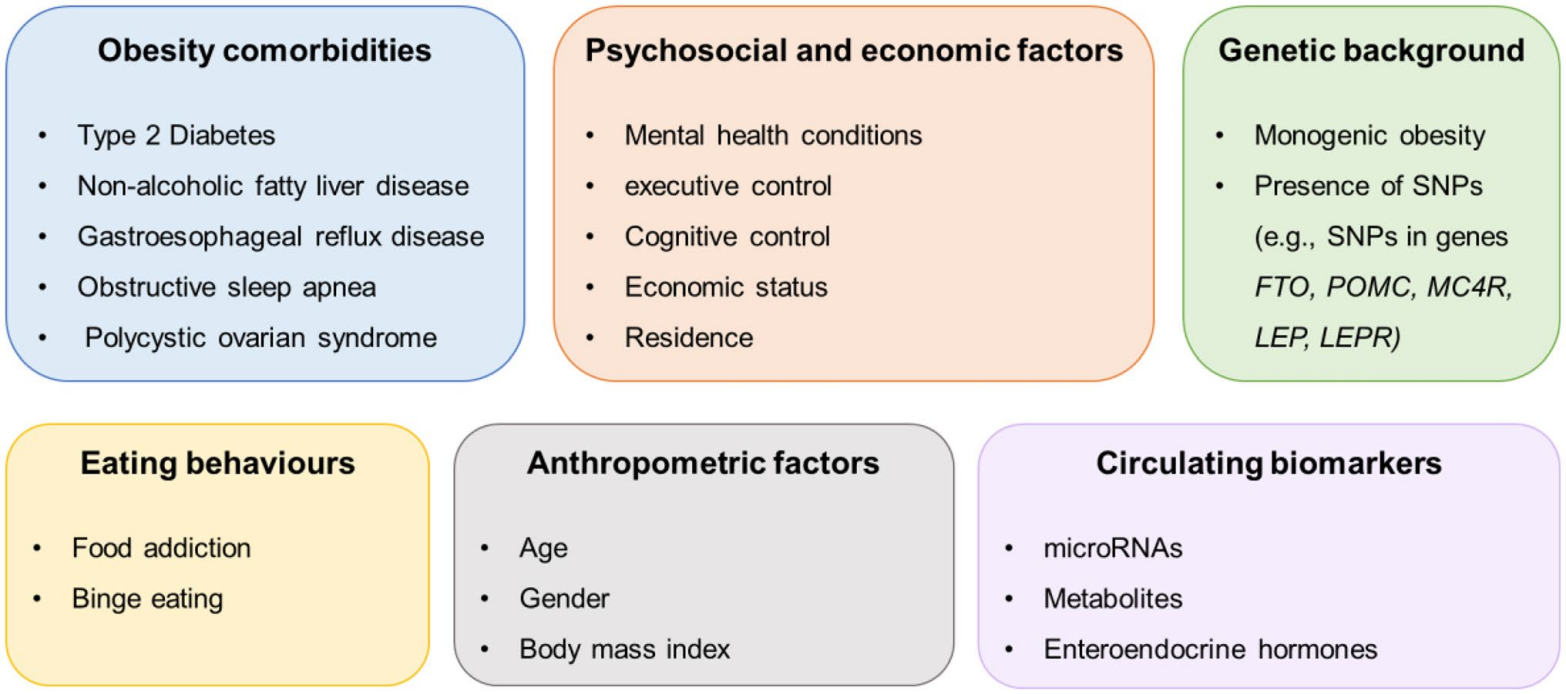
Pre-operative patient-related factors potentially associated with post-bariatric outcomes

Several reasons have been appointed as potential causes for bariatric surgery weight loss failure, however these are far from being completely understood. In this section we will address some of the pre-surgical features appointed as predictors of failure/success after bariatric surgery that may be taken in account at the time of choosing bariatric surgery, towards precision medicine in bariatric surgery prescription (Fig. 1).

### Age

Patient’s age is an important prognostic factor for several medical interventions. Numerous studies have demonstrated the safety and effectiveness of bariatric surgery in patients operated on at 70 years of age or older [24, 25]. In addition, older patients seem to experience a superior cardiovascular risk improvement [26]. Although the benefits of bariatric surgery still outweigh the risks, multiple studies found that patients age is positively correlated with higher risks of postsurgical weight regain [27–31]. Therefore, although advanced age cannot be regarded as a contraindication for bariatric surgery, elder patients will probably experience later weight regain.

Adolescents represent a small proportion of the patients undergoing bariatric surgery [32], although the figure is rapidly rising [33, 34]. The American Society for Metabolic and Bariatric Surgery Pediatric Committee recommends bariatric surgery in adolescents with a BMI > 120% of the 95th percentile with a co-morbidity or a BMI > 140% of the 95th percentile [35]. RYGB and SG were shown to lead to substantial and durable BMI loss and cardiometabolic benefits in adolescents’ [36–39]. In adolescents, the Pediatric Bariatric Study Group described 37% of BMI reduction 1 year after RYGB [40], while Inge TH *et al* reported a 26% TWL 5 years after RYGB, a weight loss magnitude similar to the one observed in adults (29%) [39]. Moreover, weight loss with lifestyle interventions before surgery was shown to predict post-surgical weight loss in adolescents [41]. So, bariatric surgery can be considered a treatment option in adolescents with obesity, as long as close post-surgical monitoring can be provided in order to ensure optimal adherence to nutritional recommendations and maximize the surgical results.

It should be noticed that approximately 7% of the patients with severe obesity with onset at pediatric age can be attributed to genetic disorders, such as Prader–Willi (PWS) and Bardet–Biedl syndromes (BBS) [42]. Literature evidence related to the success of bariatric surgery in patients with syndromic obesity is limited and mainly based on isolated case reports. Although most reports suggest that short-term weight loss can be lower than in the general population in comparison with severe obesity, bariatric surgery still leads to important metabolic benefits in those patients [43–47]. However, it should be noticed that the risk of postsurgical complications in patients with PWS is higher when compared to those of patients with non-syndromic obesity [43, 48], as well as the risk of weight regain 6 years after surgery [49]. So, there is still an unmet need to better understand how effective bariatric surgery in syndromic obesity is and which is the most appropriate surgical treatment. Noteworthy, the emergence of drugs targeting the melanocortin system that regulates satiety, such as setmelanotide, developed with the primary aim of treating patients with monogenic obesity, in the future may also open novel therapeutic alternatives for a wider patient population [50].

### Gender

There is a clear gender bias in patients undergoing bariatric surgery with a predominant number of women compared to men [51]. Additionally, males undergoing bariatric surgery have more comorbid conditions and experience more severe postoperative complications following bariatric surgery [52]. Male patients typically present for bariatric surgery at an older age, with more than double the prevalence of coronary artery disease and history of myocardial infarction compared to women [53, 54]. Bariatric surgery was demonstrated to achieve favorable outcomes in both women and men. Few studies found that there is a difference in postsurgical weight regain between the two groups, and those studies pointed towards different directions [29, 30, 55].

Greater efforts are needed to improve overall access to surgical care and narrow the gender gap.

### Pre-surgical anthropometrics

Obesity severity is usually stratified by BMI, however, other factors such as body fat distribution must be taken into account when considering prognosis of the disease. Fat distribution plays a major role in the risk of developing metabolic comorbidities, with central obesity and a raised waist circumference (WC) posing a higher comorbidity risk as compared with lower-body adiposity distribution [56].

Besides a dramatic loss of fat mass, bariatric surgery also promotes a shift in the distribution of body fat from the visceral to the subcutaneous compartment favoring metabolic improvement, associated with insulin sensitivity, and systemic inflammatory response improvement [57]. Some of these metabolic changes have been shown to be weight loss independent, particularly after RYGB, which results in more pronounced truncal fat reduction compared to SG. This differential impact upon truncal fat might in part explain why RYGB leads to greater glycemic improvement than SG despite similar weight loss, favoring the choice of this procedure in patients with established metabolic dysfunction [58, 59].

More recently, the formal indications for bariatric surgery issued in 1991 by the National Institute of Health (NIH), have been revised by the American Society of Metabolic and Bariatric Surgery (ASMBS) and the International Federation for the Surgery of Obesity and Metabolic Disorders (IFSO) to broaden the group of patients eligible. According to these guidance’s, bariatric surgery should be recommended for individuals with a BMI > 35 kg/m^2^, regardless the presence, absence, or severity of co-morbidities; should be considered in individuals with BMI of 30–34.9 kg/m^2^ who do not achieve substantial or durable weight loss or co-morbidity improvement using nonsurgical methods; individuals with metabolic disease and BMI of 30–34.9 kg/m^2^ [6]. BMI thresholds should be adjusted in the Asian population such that a BMI > 25 kg/m^2^ suggests clinical obesity, and individuals with BMI > 27.5 kg/m^2^ should be offered bariatric surgery [6].

Although the relationship between pre-surgical and post-surgical BMI is controversial, most of the studies found that baseline BMI is a significant predictor of post-surgical BMI [31, 60, 61]. Livhits *et al*, in a systematic review found a 10.1% decrease in %EWL for patients with a BMI < 50 kg/m^2^ [60]. In addition, a study enrolling 27320 patients submitted to bariatric surgery with 1 year of follow-up, found that patients with a BMI < 40 kg/m^2^ were more likely to achieve a BMI < 30 kg/m^2^ and more likely to experience comorbidity remission, when compared to patients with a BMI > 40 kg/m^2^ [61]. In what concerns postsurgical weight regain, most of the studies did not find an association between pre-surgical BMI or WC, or body fat and weight regain, although the results are not always consensual [28–30, 62, 63].

These studies results highlight the advantages of surgical management at earlier stages of obesity and that patients with a BMI under 40 kg/m^2^, are more likely to achieve a normal BMI.

### Presence of obesity comorbidities

Obesity is a risk factor for many other disorders or obesity comorbidities, which can be classified into metabolic, mechanical and mental. A few examples of these include: T2D, dyslipidemia, hypertension, non-alcoholic fatty liver disease (NAFLD), polycystic ovarian syndrome (PCOS), gastroesophageal reflux disease (GERD), obstructive sleep apnea (OSA), among many others [64, 65]. Bariatric surgery not only induces weight loss but also reduces the patients’ risk of developing obesity comorbidities and leads to a significant improvement or even remission of most of obesity comorbidities.

The unexpected possibility of achieving T2D remission was one of the most impressive achievements of bariatric surgery. The probability of undergoing T2D remission after different bariatric interventions can be influenced by both patient related factors and type of bariatric surgery procedure. Patient related factors include age, diabetes duration, family history, glycemic control and insulin treatment. Patients with younger age, shorter duration of T2D, lower preoperative glycated hemoglobin (HbA1c) and fasting blood glucose, under no insulin therapy, and without a family history of obesity were identified as being the best candidates to achieve prolonged T2D remission, independently of the surgical procedure [66]. Similarly, Stenberg E *et al* found that older age, higher HbA1c, and longer diabetes duration decreased the chances of diabetes remission after RYGB [67].

Based on the evidence of the most robust patient-related pre-operative determinants for T2D remission, predictive scores such as DiaBetter (HbA1c, T2D duration and type antidiabetic drugs) [68], DiaRem (Age, HbA1c and type of antidiabetic drugs) [69] and Ad-DiaRem (Age, HbA1c, T2D duration, number and type of antidiabetic) have been developed [70]. These models were demonstrated to have comparable accuracies for predictiving T2D remission in the short-term, with an area under the Receiver Operating Characteristic (ROC) curve (AUC) ranging 0.75 – 0.90 for RYBG [68, 70, 71]. More recently, the 5y-Ad-DiaRem score was proposed to predict medium-term T2D remission, including not only preoperative factors (HbA1c, number of antidiabetic drugs usaded, T2D duration) but also weight loss and T2D status, 1-year after surgery [72]. Of particular notice, and not surprisingly, the weight loss achieved after bariatric surgery has proved to be one of the most significant predictors for T2D remission after surgery [73].

In addition to patient related factors, the probability of achieving T2D remission is also influenced by the type of bariatric procedure. Recently, Ding L *et al*, performed a meta-analysis of RCTs (n = 70) to compare T2D outcomes after different bariatric procedures. The authors concluded that the probability of achieving T2D remission was greater in patients submitted to mini-gastric bypass when compared to those who underwent RYGB, SG, LAGB or BPD. Nonetheless, BPD was the most effective surgery at achieving long-term T2D remission [74].

Of particular notice is the fact that the relationship between T2D and post-surgical weight loss is a bidirectional one, since not only the percentage of weight loss and type of bariatric surgery influence the probability of T2D remission, but also the presence of T2D can impact on weight loss outcomes after surgery. The presence of T2D as obesity comorbidity has been reported to have a negative impact on the short and long-term weight loss and weight maintenance after bariatric surgery [18, 75].

Gestational diabetes mellitus (GDM) is associated with a near nine fold increased risk for T2D from six weeks to 28 years postpartum, as well as increased risk of metabolic syndrome and cardiovascular diseases [76]. Bariatric surgery significantly reduces the likelihood of pre-gestational and gestational diabetes, and obesity-related reproductive complications [77]. So, weight loss in women with obesity and history of GDM actively reduce the higher risk of metabolic syndrome and T2D throughout life [76, 78], so there seems that surgical techniques with proven long-lasting results, such as RYGB or DS, should be privileged. Prediabetes is also known to have a cause-effect relationship with cardiovascular disease and all-cause mortality [79]. Prediabetes often coexists with metabolic syndrome, leading to a high risk of coronary artery disease and heart failure with preserved ejection fraction [80]. Among bariatric surgery candidates, patients with prediabetes, who are at higher risk of developing T2D and related complications should be prioritized, once the largest risk reduction for macrovascular complications was seen in this group [81].

The prevalence of NAFLD increases in parallel with the prevalence of metabolic syndrome, obesity and T2D [82–84]. In patients with obesity undergoing bariatric surgery, the prevalence of NAFLD has been estimated at 91% and the prevalence of nonalcoholic steatohepatitis (NASH), at 37% [85]. All mechanisms involved at achieving weight loss and improving T2D observed after bariatric surgery also seem to play a crucial role in the amelioration or resolution of NAFLD in non-cirrhotic patients, and therefore surgery should be considered a valuable treatment option. A significant reduction of steatohepatitis and fibrosis was demonstrated to be achieved after RYGB, while after SG patients only presented significant steatohepatitis reduction [86, 87]. Despite the significant impact of bariatric surgery in NAFLD, 12.0 to 19.8% of the patients submitted to surgery are reported to develop new or worsened features of NAFLD after the procedure [88, 89]. This could be attributed to the type of procedure, degree of malabsorption and extent of malnutrition caused by bariatric surgery. Indeed, BPD was previously associated with higher liver related function morbidity, while there is extensive data to support RYGB liver safety [90, 91]. Additionally, the presence of NAFLD has been associated with lower weight loss after RYGB and mini-gastric bypass when compared with patients without this condition [92].

PCOS is the most common endocrine disorder in women at reproductive-age that is closely tied to obesity and insulin resistance, leading to multiple short-and long-term manifestations, such as oligomenorrhea, infertility, hirsutism, T2D, hypertension, dyslipidemia, and increased risk of endometrial cancer [93]. Metabolic surgery can significantly improve abnormal menstrual cycles, hirsutism, hyperandrogenism, and restore fertility in women with PCOS, and therefore should be considered a treatment option [94]. Taking into account the increased risk of metabolic syndrome in patients with PCOS, bariatric surgical techniques associated with greater improvements of metabolic dysfunction, such as RYGB or DS, might be preferentially considered, although this still requires formal demonstration.

GERD is present in up to 70% of the candidates to bariatric surgery [95]. Although the presence of GERD was not associated with the extent of weight loss after RYGB, it was shown to be associated with decreased weight loss in patients submitted to SG [95]. In addition, previous studies found that most patients undergoing RYGB have either improvement or resolution of GERD symptoms. Additionally, SG as a stand-alone bariatric procedure was associated with worsening of GERD symptoms or *de novo* reflux [95–97]. A recent meta-analysis reported rates of new onset or *de novo* reflux after SG of 23%. In addition, there was an increase of postoperative GERD after this surgical technique [98]. So, the current literature appoints the presence of GERD as a relative contraindication for SG [95, 98]. OSA is a highly prevalent obesity comorbidity. A previous study found that the risk for OSA increases 1.14 times with every unit increase in BMI [99]. Weight loss induced by bariatric surgery significantly improves OSA severity and improves OSA symptoms [100, 101]. However, there relationship between weight loss and OSA improvement is not entirely linear and a greater percentage of EWL seems to be necessary to achieve clinically relevant improvements [100].

Obesity is also associated with increased risk for cardiovascular (CV) diseases, in result of multiple mechanisms such as: the systemic effect of adipose tissue inducing cardiovascular (CV) risk factors (e.g., T2D, hypertension and dyslipidemia) and by ectopic fat deposition (e.g., myocardium and blood vessels) [102]. A recent meta-analysis showed that bariatric surgery can lead to a reduction in CV events (25–58%) and CV mortality (35–40%) [103]. Although the magnitude of weight loss is important to improve CV health, Aminian A *et al* reported that the beneficial effect of bariatric surgery was still present after adjusting for the amount of weight loss achieved, suggesting that there are additional weight-independent factors induced by bariatric surgery, which could contribute to the risk reduction for CV events [104].

Metabolically healthy patients with obesity (MHO) may have other chronic diseases, including osteoarthritis and osteoporosis, optic nerve neurodegeneration, or even hearing loss, which drastically affects patients’ life quality [105–107]. The causality relationship between optic nerve degeneration and obesity is further reinforced by the observation of improved retinal microvascular perfusion [108] and thickening of inner retinal layers, after RYGB surgery [109]. Obesity may affect hearing through obesity-related oxidative stress, inflammation, hypoxia, and death of the spiral ganglion and spiral ligament cells [110]. The risk of hearing loss was the highest among metabolically unhealthy people with obesity. MHO has a higher risk of hearing loss compared with healthy normal weight people, whereas unhealthy people with obesity had the highest risk of hearing loss, suggesting that obesity alone may increase the risk of hearing loss and unhealthy metabolic status may confer additional risk [105]. Both weight loss and improved metabolic health may be effective for hearing-loss prevention.

### Psychosocial and economic factors

**Fig. 2:**
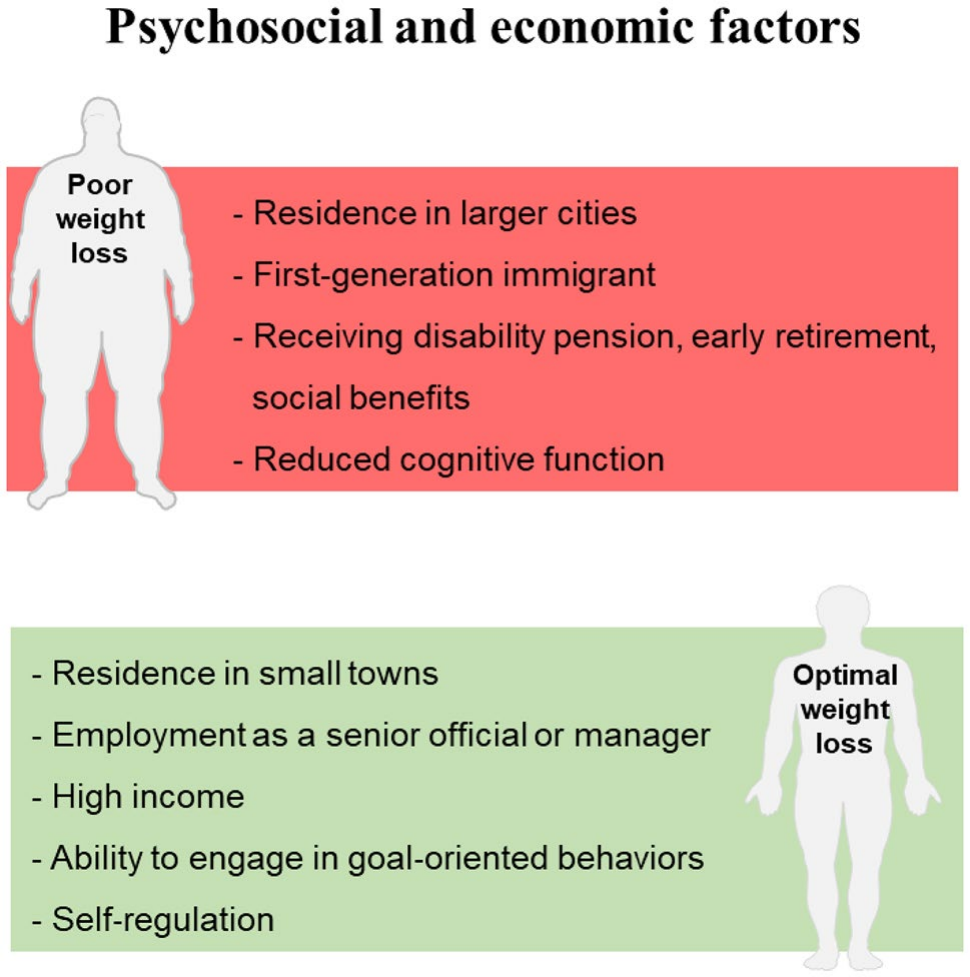
Pre-operative psychosocial and economic factors that can potentially influence post-bariatric weight loss

The prevalence of psychological distress and disorders, such as mood, personality and eating disorders, is higher in patients with obesity that seek bariatric surgery when compared to other patients with obesity [111, 112]. Previous studies found that bariatric surgery positively influenced the patient’s psychosocial status, social relationships and quality of life [112–114]. In addition, psychological disorders and alterations in executive functions (executive and cognitive control) such as the ability to engage in goal‐oriented behaviors, self‐regulation, and working memory, seems to predict weight loss outcomes in patients enrolled in lifestyle intervention programs [110, 115], while cognitive function was previously demonstrated to influence the adherence to bariatric postoperative recommendations [116] (Fig. 2).

Mental health conditions, such as depression and anxiety are frequent among patients undergoing bariatric surgery [117, 118]. Bariatric surgery was consistently associated with a decrease in the severity of depressive symptoms and in the prevalence of depression [117]. A recent multicenter study found that the prevalence of any mental disorder significantly decreased 4 years after surgery, although 7 years after RYGB there was no difference in the prevalence of mental disorders when compared with the pre-operative period [119], suggesting that long-term postsurgical psychological follow-up may be needed to maintain the short- and medium- term favorable results. Additionally, although mood disorders arising after surgery seem to be associated with weight regain after RYGB [119], the association between preoperative psychological and mental disorders and postoperative weight loss, is inconsistent [117–120].

Of note, patients submitted to bariatric surgery are at a higher risk of developing *de novo* mental, cognitive, and neurological complications derived from micronutrient deficiencies [121]. The probability of vitamin deficiencies increases with the complexity of the procedures and the magnitude of the malabsorptive component [122]. According with best practice recommendations in the absence of long-term data, lifelong multivitamin and trace element supplements are recommended for patient's after RYGB and dysabsortive procedures [123].The costs of treatment with multivitamins supplementation have always been considered a major barrier to adequate lifelong adherence [124]. The inability of the patient to comply with supplementation for economic reasons should exclude this group of patients from techniques with a greater dysabsortive component, taking into account the severity of the complications associated with vitamin deficits.

A low socioeconomic status has been reported to be associated with poorer health outcomes [125]. The data regarding the influence of socioeconomic factors on weight loss after bariatric surgery is limited. A previous study found that patients living in larger cities and being a first-generation immigrant were associated with a lower %TWL, 5 years after RYGB. In addition, subjects receiving disability pension/early retirement, social benefits, and being a first-generation immigrant, were factors associated with a lower chance of achieving a percentage of excess BMI loss (% EBMIL) ≥ 50%. Contrarily, employment as a senior official or manager, higher income, and residence in small towns were associated with a higher chance of achieving a %EBMIL ≥ 50% [126] (Fig. 2).

### Eating behaviour

Patients with severe obesity have higher rates of eating disorders compared to the general population [127, 128]. Those conditions include calories overconsumption relative to energy expenditure; picking and nibbling; binge eating; and night eating syndrome [127]. Eating disorder behaviors before bariatric surgery, in particular, binge eating, was associated with significant higher rates of depression, anxiety, substance abuse and lower health-related quality of life [127, 129]. Most of studies have reported that eating behaviors improve after bariatric surgery [127]. However, a subgroup of patients experiences a recurrence or new onset of problematic eating behaviors, in a short-term period after surgery [127, 130].

Importantly, although the relationship between the pre-operative eating behavior and post-surgical weight outcomes are not consistent, the recurrence or development of loss of control overeating behaviors or binge eating after bariatric surgery is associated with suboptimal weight loss [119, 130–133].

Food addiction is a controversial concept characterized by craving and consumption of highly palatable foods, in large amounts and in a short period of time, in compulsive eating patterns similar to those observed in other substance use disorders [134, 135]. Food addiction was recurrently associated with obesity [136, 137]. In addition, the relation between food addiction and bariatric surgery was previously investigated using the Yale Food Addiction Scale (YFAS). Although there is evidence that bariatric surgery decreases food addition, most of the studies did not find an association between preoperative food addiction and the post-surgical weight loss, independently of the bariatric procedure [138–143].

Functional magnetic resonance imaging (fMRI) is considered an important tool to understand the neurobiology of food addiction [144]. Studies using fMRI found that, compared with normal weight subjects, patients with obesity have a greater activation of neural regions associated with taste information processing, motivation, emotion and memory functions in response to high-calorie food images [145, 146]. Bariatric surgery seems to modulate the neuro-behavioral patterns since a decrease in neural response to high-calorie food images in reward related brain areas, was observed in patients submitted to bariatric surgery [147–150]. In addition, previous studies found that neural response to food cues increased in areas related to cognitive and executive control, such as orbitofrontal cortex, inferior frontal gyrus, dorsolateral pre-frontal cortex [150–152]. Pre-surgical assessment of central nervous system activation of areas related to food addiction using fMRI, was also performed in order to evaluate whether this could be used to predict post-bariatric weight loss. Holsen LM *et al* found that baseline activity in the left nucleus accumbens, a key mesolimbic reward neural region, during desire for palatable food enhancement was negatively related with the %TWL, 12 months after surgery [153]. Bach P *et al* performed a multiple regression analysis using neural and behavioral parameters associated with food addiction to predict weight loss after bariatric surgery (RYGB and SG). The authors found that individuals with high cue-induced food craving, high-perceived feeling of hunger and a low YFAS sum score were associated with a higher %TWL, 24 weeks after surgery [151]. Opposite to the long-standing assumption that food addiction could negatively influence the bariatric surgery weight loss outcomes, the available data does not point that way. Consequently, there is no evidence supporting that subjects with food addiction should be considered with caution, for bariatric surgery.

### Genetic background

Genetic background seems to be responsible for 40–75% of all the causes of obesity [154–156]. Considering this strong association, genomic studies were performed in order to identify genetic variants, in particular single-nucleotide polymorphism (SNP), that could predict weight loss after bariatric surgery. Although the results are not consistent between studies, SNPs in the genes *FTO, POMC, MC4R, LEP* and *LEPR* were recurrently investigated and associated with different weight loss trajectories after bariatric surgery [157–163]. The Swedish obese subjects (SOS) study analyzed the DNA sequence variations in 11 obesity genes (*ADIPOQ, BDNF, FTO, GNB3, LEP, LEPR, MC4R, NR3C1, PPARG, PPARGC1A* and *TNF*) and found 12 SNPs in the *ADIPOQ, FTO, LEP, LEPR, MC4R, PPARGC1A* and *TNF* gene loci nominally associated with maximum weight loss. After applying a multiple testing correction, only *FTO* SNP (rs16945088) was significantly associated with a lesser maximum weight loss after bariatric surgery [157]. In addition, when patients were evaluated in separate according to the type of bariatric surgery, this association only persisted in subjects that underwent vertical banded gastroplasty and AGB, whereas no significant association was found in RYGB subjects [157, 158]. Another study, in which selected SNPs of genes related to appetite, body composition or anthropometric measures were evaluated (*ADIPOQ, GNB3, MC4R, PPARG2, AGRP, FTO, ADRB2, POMC, ADRB3, DUSP1, IFI30, UCP2, ESR1, LEPR, APOB* and *LIPC*), found a positive association between *POMC* (rs1042571) and weight loss at 6 and 12 months after RYGB [159]. Although *ADIPOQ* (rs822396) polymorphism was also associated to weight loss 18 and 24 months after RYGB, its impact was less considerable than *POMC*. Still CD *et al* genotyped patients submitted to RYGB, for SNPs in or near the *FTO, INSIG2, MC4R* and *PCSK1* genes and found that a higher allele alterations was associated with poorer weight loss trajectories in patients with superobesity [159]. Rodrigues GK *et al*, evaluated the influence of *FTO* SNPs on weight loss and weight regain after RYGB and found that carriers of a *FTO* variant (rs9939609) presented lower %EWL at 36, 48 and 60 months after intervention. In addition, weight regain was more frequent and occurred at earlier times after surgery in the same group of patients. The *FTO* variant did not influence weight until 2 years after RYGB [164]. Similar results were observed by Balasar O *et al* that found no association between the *FTO* variant rs9939609 and %EWL at 6 months after SG [160]. Contrarily, another research group reported that the same *FTO* SNP was associated with lesser weight loss 6 and 12 months after robotic SG [165].

The data concerning the impact of *MC4R* variants on weight loss are contradictory. Mirshahi UL *et al* described that individuals carrying the *MC4R* variant rs5282087 (also known as *I251L*) achieved a lower body weight nadir when compared to individuals with the *MC4R* variant rs2229616 (also known as *V103I*) and noncarriers [161]. In contrast, other studies did not find any association between the presence of *MC4R* SNPs or mutations and the weight loss trajectories after bariatric surgery [166, 167]. A study that evaluated *FABP2, LEPR, LEP* and *FTO* polymorphisms in individuals who underwent RYGB found that the %EWL was higher in patients with obesity carriers of the *LEP* variant rs1137101, 12 and 24 months after surgery. The other genetic variants were not significantly associated with weight loss after RYGB [162]. More recently, the OBEGEN Study applied a multivariable logistic regression model that combined clinical data and SNPs to construct a clinic-genetic score for predicting weight loss after surgery. A model combining the age at surgery, type of surgery (RYGB or SG), presence of T2D, and presence of nine SNPs associated with weight loss in response to bariatric surgery (*ADIPOQ, MC4R, IL-6, PPARG, INSIG2, CNR1, ELOVL6, PLIN1*, and *BDNF*) was shown to be a good tool for predicting the weight loss response, with an area under the ROC AUC of 0.845 [163].

In the future, genetic profiling may prove to be useful for predicting patients weight loss response after surgery; however, we are still far from the time when that will become reality. Although some genetic variations were already associated with weight loss after surgery, the results are often contradictory, even for the most studied genes.

### Pre-operative circulating biomarkers

#### microRNAs

microRNAs (miRNAs) are a group of small non-coding RNA transcripts that play a key role in a large number of physiological and pathological processes [168, 169]. Multiple studies have investigated circulating miRNA signatures associated with obesity and how these change after bariatric surgery [170–174]. Consequently, studies focusing on the search for circulating miRNA associated with post bariatric clinical outcomes emerged in the last years [174–177]. However, most of these studies relied on analysis that were only performed at post-surgical timepoints or although having the pre-surgical miRNA evaluation, the miRNA profile and the clinical outcomes were correlated in the same timepoint. So, studies focusing on the identification of preoperative miRNA profiles associated with post bariatric clinical outcomes are still sparse. Recently, Yeh J *et al* identified three preoperative circulating miRNAs, involved in the regulation of the AMP-activated protein kinase signaling pathway, which were significantly correlated with weight loss after RYGB. The miR-31-5p was downregulated, while miR-328-3p and miR-181a-5p were upregulated in patients with a %EWL > 55% at 6 months after surgery, when compared with those that failed to achieve optimal weight reduction. Panels using the ratios of circulating miR-328-3p/miR-31-5p or miR-181a-5p/miR-31-5p showed a fair performance in the prediction of RYGB efficacy, with an AUC of 0.680 and 0.669, respectively [175].

So, although circulating miRNA panels harbor a great potential to recognize poor responders to bariatric surgery, the studies are limited even for the most commonly used bariatric techniques. In addition, in the future functional studies to understand the role of these miRNAs in promoting post-surgical weight loss will be needed.

#### Metabolites

Metabolomics recently emerged as a powerful tool to provide new insights on the pathological processes. Multiple studies have focused on the identification of metabolite profiles that could be used as biomarkers for diagnosis, disease monitoring and prognosis [178, 179]. Metabolomics profiling after bariatric surgery was recently reviewed by our research group, in a systematic review [180]. Amino acids, lipids, gut microbiota-related and energy-related are the metabolites that are most affected by bariatric surgery. However, the metabolite profiles were shown to be highly dependent on the type of bariatric procedure performed [180]. Preoperative metabolomic signatures associated with post‑bariatric weight loss response is far from being established. Kwon Y *et al* found that baseline levels of isoleucine and metabolites from the serotonin pathway [serotonin and 5-hydroxyindoleacetic acid (5-HIAA)] are associated with %EWL at 3 and 6 months after SG. In addition, serotonin and serotonin/5-hydroxytryptophan (5-HTrp) ratio were shown to depict a superior performance at predicting weight loss 3 months (ROC AUCs for serotonin: 0.78 and serotonin/5-HTrp ration: 0.81) and 6 months (ROC AUCs for serotonin: 0.79 and serotonin/5-HTrp ration: 0.80), after SG [181], with lower levels being associated with greater weight loss. Similar to miRNAs, metabolomics profiling although promising is far from being implemented in clinical practice to identify poor responders before surgery.

#### Enteropancreatic hormones

The anatomical gut rearrangement induced by bariatric surgery yields alterations in patients’ gut hormone profile [182, 183]. Interestingly, that profile is different according to the bariatric procedure used [182, 184–186].

Werling *et al* found that preoperative responses of glucagon-like peptide 1 (GLP-1) and peptide YY (PYY) to a mixed meal tolerance test (MMTT) do not correlate to short-term postoperative weight loss, after RYGB surgery [187]. Another study evaluated the relationship between presurgical fasting glucagon, active ghrelin, GLP-1, and glucose-dependent insulinotropic polypeptide (GIP) and maximal total body weight loss (WLmax) achieved, 5 years after RYGB, in a cohort that included patients with and without T2D. The authors found that only fasting active ghrelin was positively correlated with WLmax. Of note, not only ghrelin but also higher basal glucagon levels were correlated with greater WLmax in T2D patients. Lower levels of basal glucagon were also correlated with higher weight regain, after RYGB [27]. Higher glicentin and oxyntomodulin (OXM) levels were found to independently predict successful weight loss 18 months after RYGB and SG. No association was found between post-prandial GLP-1, PYY and ghrelin and weight loss. However, combining the postprandial changes in the levels of glicentin, GLP-1, PYY, ghrelin and OXM, the authors were able to explain 60% of the variation in weight loss 18 months after surgery [188].

Moreover, SADI-S and RYGB with a biliopancreatic limb (BPL) of 200 cm seem to elicit a favorable post-prandial glucose, GLP-1 and insulin profile, with a potentially lower risk of protein malnutrition when compared to BPD-DS and lower risk of post-prandial hyperinsulinemia as compared to RYGB with a shorter BPL [184]. The post-prandial hormone profiles observed after different bariatric surgery procedures seem to reflect the different mechanisms underlying weight loss and glucose-lowering effects and so in the future it can provide the rationale to help in the selection among bariatric surgery interventions, as long as these findings can be validated at clinical trials.

## Conclusions

Despite there are universally accepted indications for considering bariatric surgery and a vast array of surgical techniques is presented, there are no defined criteria for the use of each procedure nor guidelines for a patient-tailored decision among different technical procedures in order to optimize bariatric surgery outcomes. The decision to conduct a given procedure is empirical taking into account the individuals’ phenotype, the peri-operative risk and the surgeon experience, without any robust evidence based approach. This remains one of the most frustrating shortfalls in bariatric practice, both for clinicians and patients since weight loss after bariatric surgery is heterogeneous and can be highly unpredictable.

Although the pre-operative factors with a greater impact on post-surgical weight loss are BMI, age and T2D, these are not sufficient to explain the diversity of bariatric surgery outcomes. In addition, genetic factors and food addiction were also demonstrated to be major determinants of weight loss after surgery; while miRNAs, metabolomics and hormonal profiling, although promising are far from being robust preoperative biomarkers to be implemented in clinical practice. Consequently, there is the unmet need for a better understanding of the socio-biological drivers of weight gain, weight loss failure and weight-regain after bariatric interventions.

**Fig. 3:**
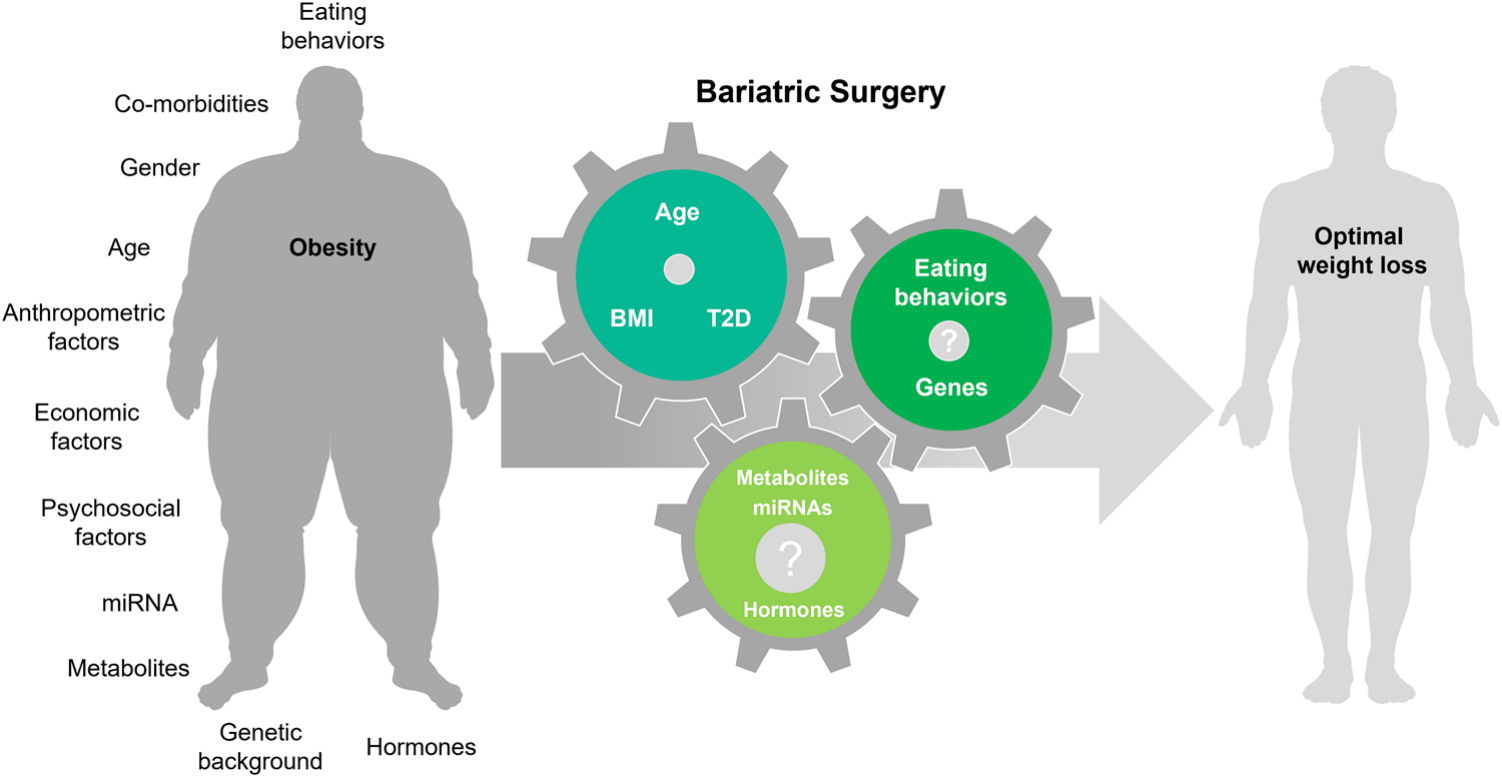
Patient related determinants that could potentially be used by artificial intelligence to predict of weight loss after bariatric surgery

Machine learning models targeting preoperative factors and effectiveness measurements of specific bariatric surgery interventions, could enable a more precise identification of the causal links between the determinants of weight loss and weight gain. Artificial intelligence algorithms grounded on preoperative factors could then be created to be used in clinical practice to predict the response to bariatric surgery interventions (Fig. 3). In a future ideal scenario, this would ultimately allow to move forward towards precision medicine in bariatric surgery prescription.

## Abbreviations

5-HIAA: 5-Hydroxyindoleacetic acid
5-HTrp: 5-Hydroxytryptophan
AGB: Adjustable gastric band
ASMBS: American Society of Metabolic and Bariatric Surgery
AUC: Area under the curve
BBS: Bardet–Biedl syndromes
BMI: Body mass index
BPD: Biliopancreatic diversion
BPL: Biliopancreatic limb
CV: Cardiovascular
DS: Duodenal Switch
EBMIL: Excess body mass index loss
EWL: Excess weight loss
fMRI: Functional magnetic resonance imaging
GDM: Gestational diabetes mellitus
GERD: Gastroesophageal reflux disease
GIP: Glucose-dependent insulinotropic polypeptide
GLP-1: Glucagon-like peptide 1
HbA1c: Glycated hemoglobin
IFSO: International Federation for the Surgery of Obesity and Metabolic Disorders
MHO: Metabolically healthy patients with obesity
miRNAs: MicroRNAs
MMTT: Mixed meal tolerance test
NAFLD: Non-alcoholic fatty liver disease
NASH: Nonalcoholic steatohepatitis
NIH: National Institute of Health
OAGB: One-anastomosis gastric bypass
OSA: Obstructive sleep apnea
OXM: Oxyntomodulin
PCOS: Polycystic ovarian syndrome
PWS: Prader–Willi syndrome
PYY: Peptide YY
RCTs: Randomized clinical trials
ROC: Receiver Operating Characteristic
RYGB: Roux-en-Y Gastric Bypass
SADI-S: Single anastomosis duodenal-ileal bypass with sleeve gastrectomy
SG: Sleeve gastrectomy
SNP: Single-nucleotide polymorphism
T2D: Type 2 diabetes
WC: Waist circumference
WLmax: Maximal total body weight loss
YFAS: Yale Food Addiction Scale
